# B cell engineering in vivo: Accelerating induction of broadly neutralizing antibodies against HIV-1 infection

**DOI:** 10.1038/s41392-022-01269-4

**Published:** 2023-01-06

**Authors:** Caijun Sun, Teng Zuo, Ziyu Wen

**Affiliations:** 1 School of Public Health (Shenzhen), Sun Yat-sen University, 518107 Shenzhen, China; 2 Key Laboratory of Tropical Disease Control (Sun Yat-sen University), Ministry of Education, 511400 Guangzhou, China; 3 Laboratory of Immunoengineering, Institute of Health and Medical Technology, Hefei Institutes of Physical Science, Chinese Academy of Sciences, 230031 Hefei, China

**Keywords:** Vaccines, Infectious diseases

In a recent study published in *Nature Biotechnology*,^1^ Nahmad and colleagues reported in vivo B-cell engineering to generate broadly neutralizing antibodies (bnAbs) against human immunodeficiency virus type 1 (HIV-1) in mice. This proof-of-concept study suggests the possibility of in vivo B-cell engineering as a novel preventive or therapeutic approach against HIV-1 infection.

**Fig. 1:**
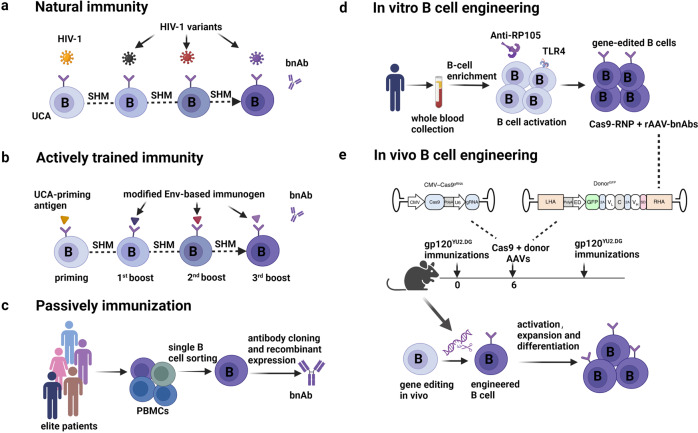
Induction of anti-HIV-1 broadly neutralizing antibodies by natural immunity, trained immunity and engineered immunity. **a** The evolution of HIV-1 mutation is much faster than that of neutralizing antibodies, and bnAbs only might be developed in elite controllers by natural immunity. **b** The lineage-based vaccine is initiated by designing appropriate UCA antigens. Once triggered, Env-based modified immunogens might further promote lineage and bind to lineage intermediates and thus promote affinity maturation for the breadth of neutralization. **c** The monoclonal antibody is isolated by single B-cell PCR (polymerase chain reaction) from PBMCs of elite controllers and can be infused into HIV-1 patients for passive vaccination treatment. The bnAb gene is integrated into Ig locus of B cells using the CRISPR-Cas9-based in vitro B-cell engineering approach (**d**) or in vivo B-cell engineering approach (**e**), respectively. UCA Unmutated common ancestor, Env envelope, bnAbs broadly neutralizing antibodies, SHM somatic hypermutation, Anti-RP105 CD180/RP105 antibody, Cas9-RNP Cas9 ribonucleoprotein, TLR4 toll-like receptor 4, PBMCs peripheral blood mononuclear cells

The epidemic of HIV-1 remains a major global health threat with ~38 million people living with HIV-1 worldwide. A safe and effective HIV-1 vaccine is highly desirable but still unavailable despite significant efforts over the past decades. One formidable obstacle in HIV-1 vaccine development is the characteristic sequence diversity and dense glycosylation of the envelop glycoprotein (Env), the sole target of neutralizing antibodies. Owing to the technical advancements in antibody-cloning methods, numerous bnAbs, which can neutralize the majority of genetically diverse HIV-1 strains, have been discovered since 2009. In contrast to neutralizing antibodies against other viruses, HIV-1 bnAbs typically originate from specific B-cell precursors and accumulate high levels of somatic hypermutation (SHM) as a consequence of continuous affinity maturation. Consistent with these unusual features of bnAbs, natural HIV-1 infection rarely elicits the production of bnAbs, with ~10–25% of infected individuals exhibiting some extent of neutralizing breadth and only 1% of patients generating highly potent bnAbs after years of infection (Natural immunity)^2,3^ (Fig. 1a).

The discovery of anti-HIV-1 bnAbs inspires two parallel directions in the development of HIV-1 vaccines: active immunization to elicit bnAbs and passive immunization with bnAbs infusion, which are two traditional strategies to achieve trained immunity against infectious diseases (Fig. 1b, c). To date, attempts to elicit bnAbs by active vaccination have only been partially successful in macaques and antibody knock-in mice with a sequential immunization of multiple immunogens. Challenges in front of this strategy include targeting the unmutated common ancestor (UCA), reproducing the longitudinal affinity maturation, and inducing improbable somatic mutations in antibody sequences.^4,5^ Alternatively, bnAb infusion has been highly successful in preventing infection in macaques and humanized mice, and the prevention efficacy in humans is under clinical evaluation. Moreover, bnAbs infusion has achieved a remarkable therapeutic effect in viremic individuals and prolonged suppression of viral rebound after interruption of antiretroviral therapy. Despite this promising efficacy, passive vaccination with bnAbs faces several challenges including virus escape, antibody durability, and manufacturing cost.

To overcome these challenges of bnAb-based vaccine, several groups developed an in vitro B-cell engineering approach, in which they integrated bnAb genes into Ig locus of B cells using CRISPR-Cas9 (Fig. 1d). When adoptively transferred to recipients, the edited B cells were able to participate in humoral immune responses and secrete the corresponding bnAbs, implying the potential of B-cell engineering as a novel strategy against HIV-1 infection. However, clinical translation of this approach is complex and cost-intensive, since sophisticated technical protocols, major histocompatibility complex compatibility, and specialized medical centers are required. To further address the challenges related to in vitro B-cell engineering, Nahmad and colleagues developed this approach of in vivo B-cell engineering.^1^ During the immunization with HIV-1 antigen gp120, a single injection of two adeno-associated viral (AAV) vectors was administrated intravenously in mice. Specifically, one AAV vector encodes the *Staphylococcus aureus* Cas9 (saCas9), single-guide RNA (sgRNA) targeting immunoglobulin heavy chain (IgH) locus, and the other one encodes an anti-HIV-1 bnAb 3BNC117. Of note, B cells were effectively gene-edited in vivo, and the engineered B cells were involved in the germinal center reaction and differentiated into plasma cells to secrete a high titer of 3BNC117 in mice. To increase the safety of this strategy, they also separated the saCas9 and sgRNA between the two AAVs, and further constructed the saCas9 sequence under a B-cell-specific CD19 promoter to prevent off-target cleavage in undesired tissues. Compared to natural immunity and trained immunity, this strategy of in vivo B-cell engineering is more simple, fast, and cost-effective, and can be interpreted as a novel combination of passive immunity and active immunity, so-called “engineered immunity” (Fig. 1e).

This strategy has the potential to treat infectious diseases as well as non-communicable conditions, such as cancer and autoimmune disease. However, further confirmation and optimization might be required for its future clinical applications: (1) This study showed that a high titer of 3BNC117 was generated in mice after in vivo B-cell engineering, but the protective efficacy should be further tested in vivo in HIV-1-infected individuals; (2) To potentially avoid immune evasion, combining a variety of complementary bnAbs may be necessary for long-term control of HIV-1, but how to engineer B cells in vivo to generate multiple bnAbs will be a challenge; (3) To improve the safety of this approach, novel delivery systems, such as mRNA platform, can be adopted in the future.
